# The Arthroscopic Biceps Rerouting Technique Shows Better Early Clinical Outcomes within 1 Year Than Partial Repair in Large to Massive Rotator Cuff Tears

**DOI:** 10.3390/medicina60020240

**Published:** 2024-01-30

**Authors:** Hyung-Seok Park, Kwon-Hyoung Ham, Yang-Soo Kim

**Affiliations:** 1 Department of Orthopedic Surgery, Chosun University Hospital, Gwangju 61453, Republic of Korea; hyungseok929@chosun.ac.kr (H.-S.P.); gu188b@hanmail.net (K.-H.H.); 2 Department of Orthopaedic Surgery, College of Medicine, Chosun University, Gwangju 61452, Republic of Korea; 3 Department of Orthopedic Surgery, Seoul St. Mary’s Hospital, The Catholic University of Korea, Seoul 14662, Republic of Korea

**Keywords:** rotator cuff tear, arthroscopic partial repair, arthroscopic biceps rerouting

## Abstract

*Background and Objectives*: The arthroscopic biceps rerouting (BR) technique was introduced as a novel technique for large-to-massive rotator cuff tears (LMRCTs). This method can restore shoulder stability, and its biomechanical effect was verified in a cadaver experiment. Recent reports have shown favorable clinical outcomes. This study aimed to compare the clinical outcomes of partial repair with those of the BR technique in LMRCTs. *Materials and Methods*: We included 72 patients who had undergone LMRCTs. Arthroscopic partial repair was performed in 31 patients (group I), while repair with the BR technique was performed in 41 patients (group II). Functional and imaging outcomes were evaluated and compared using a visual analog scale for pain, range of motion (ROM), American Shoulder and Elbow Surgeons (ASES) score, Constant score, and Korean Shoulder Score (KSS) before the surgery and at 3, 6, and 12 months after the surgery and at the last follow-up. Tendon integrity was investigated using magnetic resonance in all the patients at 12 months postoperative. *Results*: The mean follow-up period was 22.11 ± 10.47 months (range, 12–53 months). Forward flexion (*p* = 0.02) and external rotation at the side (*p* = 0.04) were significantly higher in group II than in group I on postoperative day (POD) 12 months. For internal rotation, group II showed significant improvement compared to group I on POD 3 months (*p* = 0.002) and at 6 months postoperative (*p* = 0.01). On POD 12 months, the ASES (*p* = 0.047) and KSS (*p* = 0.048) were better in group II. Significant improvements in pain and clinical scores were observed in both groups at the last follow-up. However, the two groups had no significant differences in pain, clinical scores, or ROM at the last follow-up. Retear of the repaired tendon was observed in nine patients in group I (29.03%) and eight in group II (19.51%, *p* = 0.35). *Conclusions*: Partial repair and repair using the BR technique effectively improved clinical and radiologic outcomes. The BR technique revealed early recovery of ROM and better clinical results than partial repair at one year postoperative. This could be a useful method in treating LMRCTs.

## 1. Introduction

Large to massive rotator cuff tears (LMRCTs) are challenging to manage, especially in achieving satisfactory healing. They are almost always accompanied by chronic tendon wear, severe retraction, muscle atrophy, and fatty degeneration [1,2,3]. Among the numerous repair techniques for LMRCTs, Burkhart’s was the first to introduce partial repair [4]. Its basic principle is maintaining a force couple that enables sufficient shoulder function, leading to functional repair, not anatomical repair of the part [5]. Partial repair is relatively simple compared with other complex techniques, and good results have been reported [6]. Although partial repair is not a perfect solution, it is a treatment technique recognized to produce somewhat favorable results among the existing methods; surgeons might consider it a viable option. However, a recent study showed that almost half of patients were dissatisfied with the worsening results over time, even though partial repair could improve selected outcomes at a two-year follow-up [7,8].

In recent years, arthroscopic biceps rerouting (BR) has been introduced as a novel technique for large irreparable rotator cuff tears, and a cadaveric study reported that it was effective in providing biomechanically valid results [8]. A few studies have introduced the use of the long head of biceps tendon (LHBT) for treating massive rotator cuff tears [9,10,11,12,13]. Surgical methods utilizing the LHBT can be broadly categorized into biceps augmentation, anterior cable reconstruction, and BR. Biceps augmentation primarily aims to fill defects following rotator cuff repair. In contrast, anterior cable reconstruction focuses on reconstructing the anterior cable of the rotator cuff, aiming to rebuild an essential structural component of the rotator cuff. In other studies, the LHBT was tenotomized distally, and the patient was at risk of having unexpected disadvantages of biceps tenotomy [11,12,13]. In contrast, Kim et al. [14] proposed a novel technique called “arthroscopic biceps rerouting” (BR) to treat LMRCTs that maintain the original continuity of the LHBT. With the BR technique, the LHBT is rerouted to a new groove posterior to the original groove without distal tenotomization. Several distinctions set BR apart from other methods that utilize the LHBT. Initially, BR involves the utilization of the LHBT, which is freed from its surrounding soft tissues, including the transverse humeral ligament that encircles the LHBT in the biceps groove, without severing it. Furthermore, before the repositioning and fixation of the LHBT at its new location on the humeral head, it is necessary to create a fresh groove at the footprint’s center specifically for attachment of the LHBT, achieved through burring. Subsequently, the repositioned LHBT is anchored into this newly established groove using two anchors, positioned medially and laterally, to ensure its security. Recent studies have reported favorable preliminary outcomes [8,15]. This study aimed to compare the clinical and radiological outcomes between the two techniques. We hypothesized that BR would show better clinical and radiological outcomes than partial repair in LMRCTs.

## 2. Materials and Methods

### 2.1. Inclusion and Exclusion Criteria

This retrospective comparative study of partial repair and BR performed by a senior surgeon at a single institution was conducted between December 2013 and June 2019. The lesions were checked preoperatively using magnetic resonance imaging (MRI) and visualized arthroscopically during surgery. The indication for surgery was a rotator cuff tear that did not respond to medication and physiotherapy for at least six months.

Inclusion criteria were as follows: 

(1) Patients who underwent partial rotator cuff repair with the articular surface of the humeral head exposed, that is, footprint coverage of <80% [16].

(2) Patients who with follow-up for at least one year after surgery who underwent MRI preoperatively and one year postoperatively.

Patients with a history of trauma, labral or biceps lesions, glenohumeral arthritis, concomitant shoulder stiffness, or subscapularis tears requiring repair were excluded. This study was approved by the Institutional Review Board of our hospital (No. CHOSUN 2021-08-001-001).

### 2.2. Baseline Characteristics

In total, 96 patients underwent partial repair or BR. Nineteen patients were lost to follow-up one year after surgery, and five patients declined participation in the study. Finally, 72 patients were included in this study. Group I (*n* = 31) included patients who underwent partial rotator cuff repair without any LHBT procedures. Group II (*n* = 41) included patients who had undergone BR. There was no specific selection method for the surgical procedures. Since March 2017, BR has been performed in patients with similar conditions in the absence of biceps long head tendon lesions.

### 2.3. Surgical Technique

One senior surgeon performed all the operations at a single institute. All the arthroscopic procedures were performed with the patient under general anesthesia in the lateral decubitus position. We used a standard posterior portal to check the glenohumeral joint in order to address associated intra-articular lesions. An anterior portal was routinely created through the rotator interval for the working portal using a spinal needle. A probe was used to check for the presence of the LHBT. Arthroscopic debridement was performed on partial-thickness subscapularis tears less than the superior third. The subacromial space was examined using a posterior viewing portal. An anterolateral working portal was created by using a spinal needle. Debridement, bursectomy, and acromioplasty were also performed. We switched the viewing portal to a posterolateral rear-viewing portal and checked the tear size and reducibility of the tendon.

In group I, partial repair was performed if excessive tension occurred to cover the footprint, even after sufficient tendon release. A modified Mason–Allen single-row or transosseous-equivalent technique was used. It was performed considering tendon tear geometry, tissue quality condition, and force couple.

**Figure 1:**
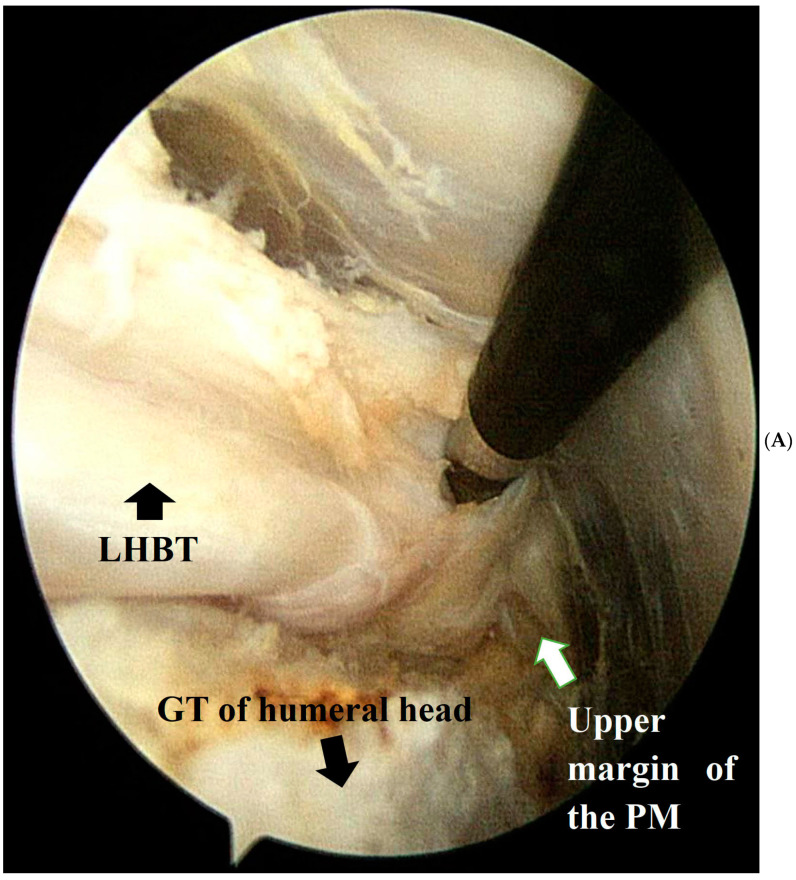

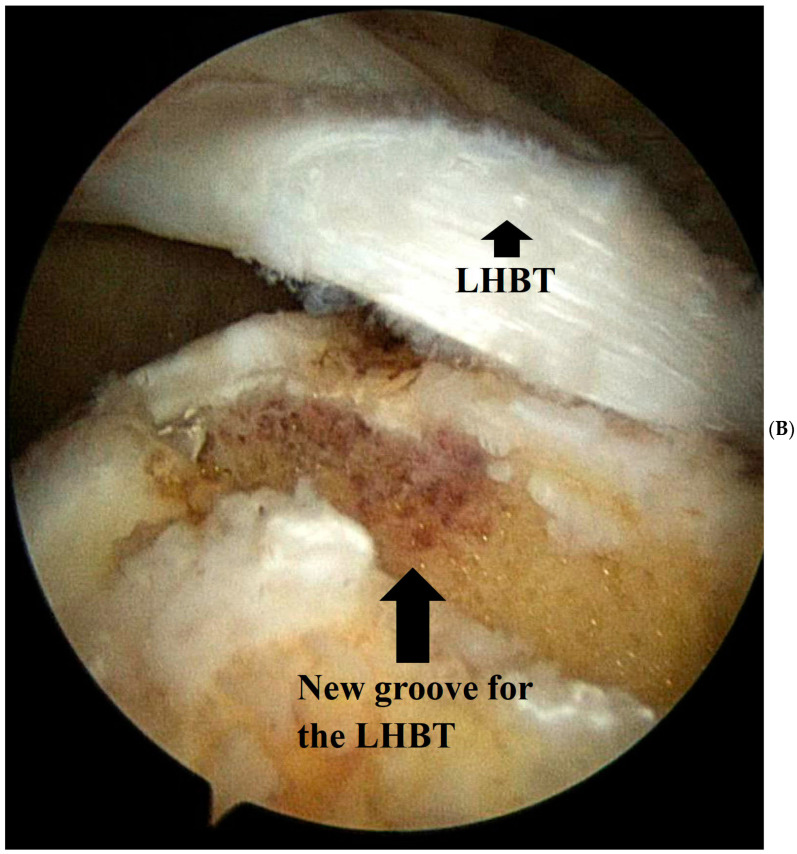

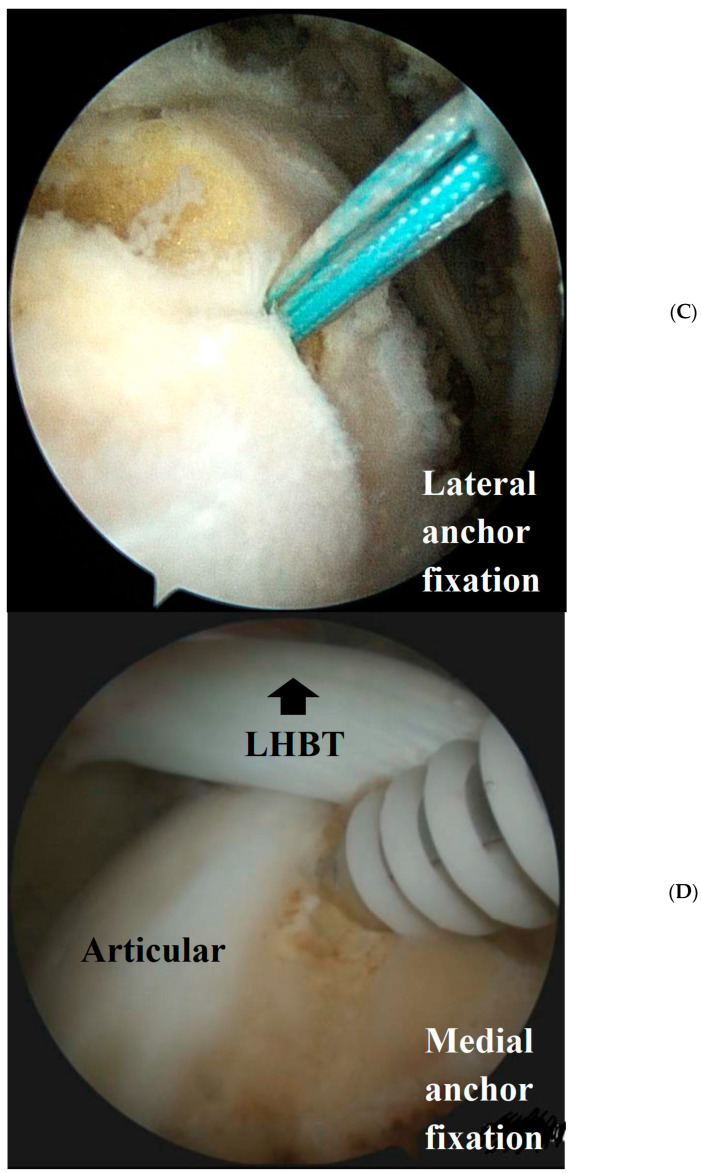
Subacromial space of right shoulder viewing from the posterior portal with left side down lateral decubitus position. (**A**) Soft tissue removal around the LHBT to make its mobility until the upper margin of the PM. (**B**) A new groove was placed posterior to the original groove of the LHBT. (**C**,**D**) Medial and lateral anchor fixation for rerouted LHBT. (LHBT, long head of biceps tendon; GT, greater tuberosity; PM, pectoralis major).

The surgical techniques in group II (cases of BR) are described in detail in the arthroscopy techniques paper [14]. The soft tissues around the LHBT were released to the upper margin of the pectoralis major, which was located approximately 6 cm distal to the anterolateral portal (Figure 1A). We created a new groove to place the released biceps tendon by using an elliptical burr (Figure 1B). Two anchors were used to fix the biceps tendon to the new groove. One anchor was inserted at the lateral margin of the newly created biceps groove. Another anchor was placed in the medial part of the rerouted biceps tendon at the cartilage margin (Figure 1C,D). Lateral and medial ties at each side were performed to fix the rerouted biceps tendon on the newly made groove. One or more anchors were used to repair the rotator cuff tendon.

### 2.4. Clinical and Radiological Evaluation

The American Shoulder and Elbow Surgeons (ASES) score, Korean Shoulder Score (KSS), and Visual Analog Scale (VAS) pain score (0–10) were used to assess clinical outcomes. A goniometer measured the range of motion (ROM) of forward flexion (FF), external rotation at the side, and external rotation at 90° of abduction. Internal rotation was defined as the highest vertebral level at which the tip of the thumb could reach a sedentary position. The vertebral levels were numbered serially from below the sacrum (level 0) to the seventh thoracic vertebra (level 12). Clinical evaluations, including ROM, were performed before surgery, 3, 6, and 12 months after surgery, and at the last follow-up. Radiological evaluation was performed using plain anteroposterior radiographs and MRI. The acromiohumeral interval (AHI), which is the smallest distance from the superior margin of the humeral head to the undersurface of the acromion, was checked preoperatively and three months postoperatively. Magnetic resonance imaging was performed 12 months postoperatively. We used the Sugaya classification [17] to evaluate the repaired cuff tendon and considered Sugaya classification types 4 and 5 as retears. Clinical and radiological evaluations were performed by a physician blinded to the study.

### 2.5. Rehabilitation

All the patients underwent similar rehabilitation protocol after the surgery. An abduction brace was applied for six weeks after the surgery. After the brace was discarded, the patients were taught the sleeper-stretch and pulley exercises. Two months after the surgery, the patients started pendulum exercises. Three months after the surgery, the patients underwent muscle-strengthening exercises using Theraband. After six months, the patients were able to engage in preoperative and sports activities.

### 2.6. Statistical Analysis

All statistical analyses were performed using SPSS (version 24.0; SPSS Inc., Chicago, IL, USA). A paired *t*-test (two-tailed *t*-test) was used to compare ROM and functional scores at each time point between the two groups. The Mann–Whitney U-test was used to compare the integrity of the repaired cuff tendon with the Sugaya classification. Statistical significance was set at *p* < 0.05.

## 3. Results

Demographic parameters showed no significant differences between the two groups (*p* > 0.05, Table 1). Group I included 10 males and 21 females with a mean age of 67.1 years (range, 45–77 years). Group II included 15 males and 26 females with a mean age of 65.5 years (range, 54–82 years). The mean follow-up period was 23.25 (range, 12–53 months) in group I and 21.24 months (range, 12–35 months) in group II. In Group I, there was a significant improvement in the clinical outcomes preoperatively and at the last follow-up (VAS, 3.57 ± 1.87 to 1.57 ± 0.98; ASES score, 55.86 ± 13.38 to 79.94 ± 11.17; CS, 62.50 ± 16.38 to 81.56 ± 11.25; and KSS, 60.73 ± 14.51 to 81.31 ± 16.87; *p* < 0.001), and there was no significant difference in ROM (*p* > 0.05) (Table 2). Similarly, in group II, there was a significant improvement in clinical outcomes preoperatively and at the last follow-up (VAS, 2.96 ± 2.05 to 1.33 ± 1.34; ASES score, 54.67 ± 22.33 to 82.83 ± 11.94; CS, 60.08 ± 19.18 to 82.94 ± 7.17; and KSS, 53.38 ± 19.71 to 85.69 ± 10.21; *p* < 0.001), but there was no significant difference in ROM (*p* > 0.05) (Table 3).

**Figure 2:**
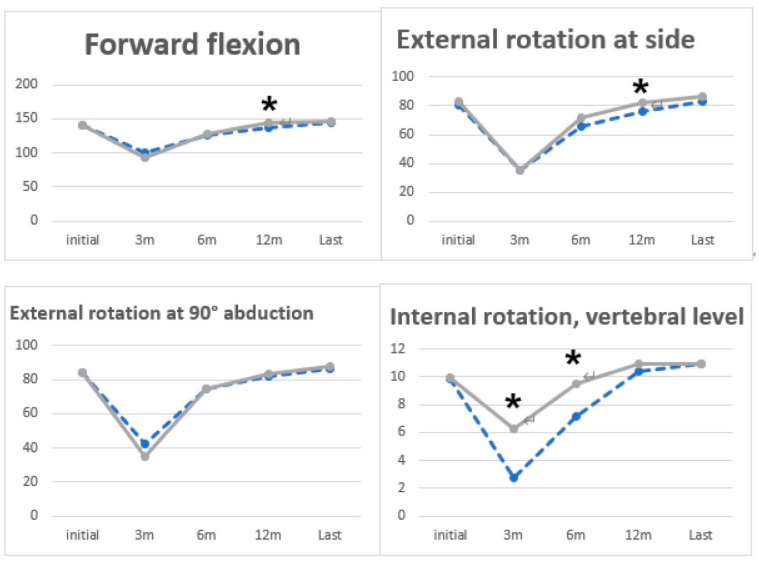
The vertebral level was numbered serially as follows: 0 point for any level below the sacral region and 1 point for each level above the sacrum (dotted line: Group I, solid line: Group II, *: statistical significance).

**Figure 3:**
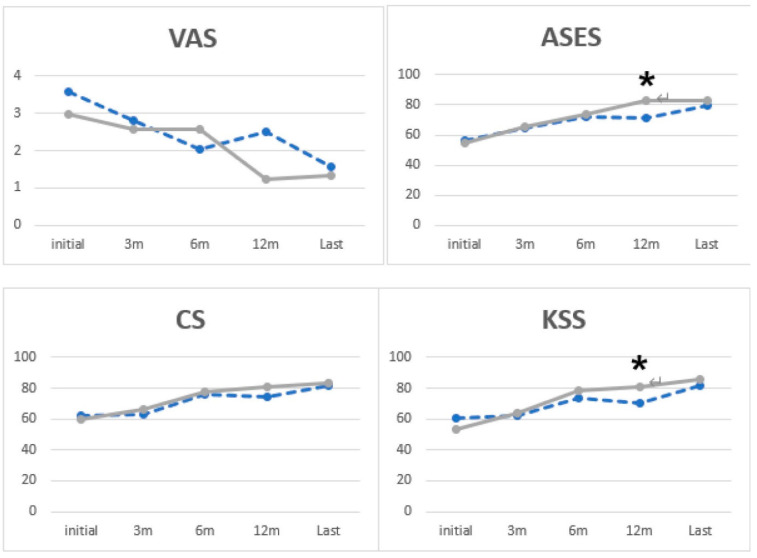
VAS, visual analog scale; ASES, American Shoulder and Elbow Surgeons; CS, Constant Score; KSS, Korean Shoulder Score (dotted line: Group I, solid line: Group II: *: statistical significance).

In group I, compared to group II, FF (*p* = 0.02, group I: 137.24 ± 9.87 and group II: 143.79 ± 8.09, Figure 2 and Table 4) and external rotation at the side (*p* = 0.04, group I: 75.71 ± 12.60 and group II: 82.14 ± 6.86; Figure 2 and Table 4) at 12 months postoperatively and internal rotation on POD 3 months (*p* = 0.002, group I: 2.74 ± 2.99 and group II: 6.26 ± 3.37; Figure 2 and Table 4) and at 6 months (*p* = 0.01, group I: 7.16 ± 3.33 and group II: 9.48 ± 2.26; Figure 2 and Table 4) showed significantly better ROM. There was no significant difference in ROM between the two groups at the last follow-up (*p* > 0.05). There were significantly better clinical outcomes in ASES (*p* = 0.047, group I: 71.25 ± 21.72 and group II: 83.13 ± 12.89) and KSS (*p* = 0.048, group I: 70.60 ± 15.72 and group II: 80.67 ± 14.30) at 12 months postoperative (Figure 3 and Table 5).

We used the Sugaya classification system to assess the integrity of the repaired cuff tendon. Type IV or V was considered a retear. Retear was found in nine patients in group I (29.03%, 9/31) and eight in group II (19.51%, 8/41, *p* = 0.35; Table 6). Rupture of the LHBT was confirmed in six of eight cases of retears in group II. A long head of biceps tendon rupture around the medial anchor was observed in five cases, and rupture around the lateral anchor was observed in one case. Nevertheless, Popeye deformity was not observed, and none of the patients complained of upper arm pain related to the bicep tendon. In both groups, no significant difference in AHD or the occurrence of glenohumeral joint osteoarthritis was observed during the follow-up period.

## 4. Discussion

In our study, both the partial repair and BR groups showed significant improvements in clinical outcomes of LMRCTs. The BR group showed an earlier recovery of ROM after the surgery than the partial repair group, and it showed significantly better results in internal rotation at three and six months postoperative and FF and external rotation at twelve months postoperative. Additionally, clinical scores, such as the ASES and KSS, were significantly better in the BR group than in the partial repair group at 12 months postoperative. This suggests that the BR group recovered faster than the partial repair group with respect to ROM and clinical outcomes.

The retear rate in the partial repair group was higher than that in the BR group, although there was no statistically significant difference between the two groups. Additionally, the BR group gave more weight to overall good results in the Sugaya classification than the partial repair group. There was no rupture of the LHBT in well-healed patients in the BR group. Long head of bicep tendon rupture was observed in the retears of patients with repaired cuff tendons. We can deduce that the rerouted LHBT is closely related to the cuff healing process. A recent animal study showed that the rerouted LHBT had increased load-bearing strength compared to the native superior capsule. It was also confirmed that the rerouted LHBT could engraft into the new groove for the LHBT and show excellent biomechanical performance [18]. Histopathological studies also showed that morphological changes in the rotator cuff tendon were positively correlated with the degree of LHBT degeneration [19].

Partial repair is a relatively simple and easy technique that restores the force couple by replacing a non-functional tear with a functional tear. Numerous clinical and biomechanical studies have been conducted on this technique [2]. Kim et al. reported a significant improvement after arthroscopic partial repair in patients diagnosed with LMRCTs, and 81% of the patients who underwent surgery were satisfied [6,20]. A study by Yoo et al. [16] showed that approximately 50% of patients showed deterioration in clinical scores and poor satisfaction in long-term follow-up. Additionally, a decrease in AHI was observed in patients with exacerbation. Similarly, another study confirmed a decrease in AHI at a long-term follow-up after partial repair [21]. Many recent reports have argued that partial repair showed good results at short-term follow-up, but there is a possibility that it may worsen over time, such as a decrease in AHI. Additionally, two studies showed relatively low healing quality and high retear rate when partial repair was performed [22,23].

Recently, BR has been introduced as an innovative method for the treatment of LMRCTs [14]. After creating a new groove for the LHBT at the mid-portion of the footprint, the LHBT is moved to the new groove and the remaining rotator cuff is repaired. According to the preliminary outcomes of 61 patients who underwent BR followed up for over 18 months, good results were found for pain VAS, ASES, and KSS. The acromiohumeral interval increased from 7.1 mm to 9.7 mm, and 16 (26%) patients showed retear of the repaired rotator cuff [24]. Most recently, there was a study comparing BR and conventional repair, and both groups showed good clinical results after surgery, but there was no significant difference between the two groups. However, the retear rate in the BR group was 18.6% and 48.1% in the conventional repair group, showing a significant difference between the two groups [15].

We can postulate several possible reasons why the BR group showed better results in the early postoperative period than the partial repair group did. One reason is that the proximal portion of the biceps in the intra-glenohumeral joint can biomechanically act like the superior capsule of the glenohumeral joint [24]. In our previous cadaveric biomechanical study, BR showed a significant decrease in superior humeral translation and reduced subacromial contact pressure compared with the partial repair group [8]. Arthroscopic biceps rerouting stabilizes the glenohumeral joint, which reduces the load on the repaired tendon and lowers tension to facilitate blood supply. It also reduces acromial contact pressure, thereby reducing the stress on the repaired tendon. Ultimately, this provides a suitable environment for tendon healing. This can promote reinforcement of the repaired cuff and are thought to serve as an internal splint during the critical period of cuff healing. In addition to these mechanical effects, the use of biceps without cutting positively affected the blood supply required for cuff healing [15]. Furthermore, creating a new groove for the biceps, which is the core technique of BR, helps cuff healing by creating materials, such as stem cells from the bone marrow, fibroblasts, and chemical mediators, such as various growth factors and cytokines for tissue healing. According to a recent animal study, the rerouted biceps tendon can be remodeled progressively. Over time, it heals into new grooves in the tendon, resulting in bone–tendon healing that improves biomechanical performance [18]. According to another study, the LHBT is an important pain generator in the shoulder, and failure to properly treat the LHBT during cuff repair leads to impaired functional results. It is possible that when BR is performed, treatment for the LHBT is carried out simultaneously, resulting in good results [25].

The tenodesis effect of BR is thought to be similar to that of biceps tenodesis. If the distal part is cut and a tenotomy is performed after all BR procedures, it will show a similar effect as the biceps tenotomy. Arthroscopic biceps tenotomy, especially when combined with a rapid rehabilitation protocol, yields promising outcomes in reducing shoulder pain and facilitating a return to previous activities, even in older patients [26]. The approach to managing LHBT tendinopathy should be individualized, considering both the potential for cosmetic deformity and the specific needs and activities of the patient. The recent study concludes that both biceps tenodesis and tenotomy result in similar improvements in patient-reported and functional outcomes for treating the LHBT or SLAP pathology. However, tenotomy has a higher association with cosmetic deformity [27].

The study is retrospective in nature, conducted at a single center, and a single surgeon performed all procedures. These factors may limit the generalizability of the findings. The outcomes assessed are short-term; the study does not evaluate long-term outcomes. However, follow-up for further assessment is ongoing. The rerouting technique for the LHBT was experimental, with the tendon repositioned posteriorly. The long-term effects of this technique remain unknown. There was no detailed tracking of the exact number of anchors used or their specific locations. Additionally, comorbidities such as smoking status were not recorded. While a reduced retear rate was observed post-procedure, the study lacks comprehensive data to elucidate the biological or biomechanical reasons for this outcome fully. One significant limitation of our study stems from the timeline of patient inclusion and the subsequent introduction of the BR technique. Patient enrollment began in 2013, but the BR technique was only adopted in 2017. This gap introduces a potential learning curve for the surgical team, which could have influenced the outcomes for patients undergoing the procedure after its introduction. Additionally, this delay meant that patients who received partial repairs before implementing the BR technique had a more extended follow-up period than those treated with the BR technique. Moreover, there appears to be a selection bias in applying the BR technique, likely influenced by the severity of the rotator cuff tears. Shoulders with lower-severity tears were more frequently treated using the BR technique. In contrast, the BR technique was often deemed inapplicable in cases with high-grade tears that were not sufficiently mobile due to the absence of viable tendon tissue. This selection bias could skew the comparison of clinical outcomes between patients treated with traditional methods and those treated with the BR technique. These factors combined suggest that the differences in follow-up duration and the selection criteria for the BR technique may have impacted the study’s findings, making it challenging to draw definitive conclusions regarding the efficacy of the BR technique across all severities of shoulder tears.

## 5. Conclusions

Both partial repair and repair with the BR technique for LMRCTs were effective in improving clinical and radiologic outcomes at the short-term follow-up. However, the BR technique yielded early recovery of ROM and better clinical outcomes than partial repair at one year postoperative. Retear occurred at a higher probability in group I than in group II. This may be a useful method for repairing LMRCTs.

## Figures and Tables

**Table 1:** Preoperative demographic data.

	Group I (*n* = 31)	Group II (*n* = 41)	*p* Value
Age, years	67.10	65.49	0.87
Sex, male/female, *n*	10/21	15/26	0.69
Follow-up period (range), months	23.25 (12–36)	21.24 (12–34)	0.93
Tear size, mm			
medial to lateral	31.9	31.3	0.14
anterior to posterior	26.1	27.4	0.71
Acromiohumeral interval	7.89	7.91	0.83
Fatty infiltration (Goutallier classification), *n* (%)			0.13
Grade 1	0 (0)	0 (0)	
Grade 2	4 (14.8)	10 (11.1)	
Grade 3	19 (59.2)	25 (66.6)	
Grade 4	8 (25.9)	6 (22.2)	
Initial ROM			
Forward flexion, °	140.38 ± 9.89	140.19 ± 18.35	0.97
External rotation at side, °	80.38 ± 10.76	82.69 ± 14.02	0.52
External rotation at 90° of abduction, °	83.85 ± 7.52	84.23 ± 13.01	0.90
Internal rotation, vertebral level	9.88 ± 2.79	9.92 ± 2.81	0.96
Initial clinical score			
VAS score for pain	3.57 ± 1.87	2.96 ± 2.05	0.06
ASES score	55.86 ± 13.38	54.67 ± 22.33	0.80
Constant score	62.50 ± 16.38	60.08 ± 19.18	0.49
Korean Shoulder Score	60.73 ± 14.51	53.38 ± 19.71	0.24

**Table 2:** Comparison of outcomes after partial repair.

	Preoperative	Last Follow-Up	*p* Value
Range of motion			
Forward flexion, °	140.38 ± 9.89	145.18 ± 6.57	0.48
External rotation at side, °	80.38 ± 10.76	82.92 ± 9.99	0.56
External rotation at 90° of abduction	83.85 ± 7.52	86.54 ± 6.29	0.21
Internal rotation, vertebral level	9.88 ± 2.79	10.95 ± 1.39	0.08
Clinical outcome			
VAS	3.57 ± 1.87	1.57 ± 0.98	<0.001
ASES score	55.86 ± 13.38	79.94 ± 11.17	<0.001
CS	62.50 ± 16.38	81.56 ± 11.25	<0.001
KSS	60.73 ± 14.51	81.31 ± 16.87	<0.001

**Table 3:** Comparison of outcomes after biceps rerouting.

	Preoperative	Last Follow-Up	*p* Value
Range of motion			
Forward flexion, °	140.19 ± 18.35	146.48 ± 6.32	0.15
External rotation at side, °	82.69 ± 14.02	86.67 ± 6.37	0.16
External rotation at 90° of abduction	84.23 ± 13.01	87.69 ± 5.14	0.63
Internal rotation, vertebral level	9.92 ± 2.81	10.95 ± 2.01	0.09
Clinical outcome			
VAS	2.96 ± 2.05	1.33 ± 1.34	<0.001
ASES score	54.67 ± 22.33	82.83 ± 11.94	<0.001
CS	60.08 ± 19.18	82.94 ± 7.17	<0.001
KSS	53.38 ± 19.71	85.69 ± 10.21	<0.001

**Table 4:** Range of motion at each time point.

	Preoperative	3 Months	6 Months	12 Months	Last Follow-Up
Forward flexion, °					
Group I	140.38 ± 9.89	99.82 ± 22.21	126.72 ± 15.59	137.24 ± 9.87	145.18 ± 6.57
Group II	140.19 ± 18.35	92.07 ± 29.20	128.44 ± 19.04	143.79 ± 8.09	146.48 ± 6.32
*p* value	0.97	0.25	0.69	0.02	0.47
External rotation at side, °					
Group I	80.38 ± 10.76	35.20 ± 21.63	66.00 ± 17.93	75.71 ± 12.60	82.92 ± 9.99
Group II	82.69 ± 14.02	35.60 ± 23.11	72.00 ± 12.43	82.14 ± 6.86	86.67 ± 6.37
*p* value	0.52	0.95	0.13	0.04	0.19
External rotation at 90° of abduction					
Group I	83.85 ± 7.52	42.40 ± 23.50	74.84 ± 14.35	81.79 ± 8.63	86.54 ± 6.29
Group II	84.23 ± 13.01	35.20 ± 22.94	74.52 ± 12.34	83.57 ± 9.89	87.69 ± 5.14
*p* value	0.90	0.30	0.92	0.50	0.50
Internal rotation, vertebral level					
Group I	9.88 ± 2.79	2.74 ± 2.99	7.16 ± 3.33	10.43 ± 1.86	10.95 ± 1.39
Group II	9.92 ± 2.81	6.26 ± 3.37	9.48 ± 2.26	10.90 ± 1.61	10.95 ± 2.01
*p* value	0.96	0.00	0.01	0.45	1.00

**Table 5:** Clinical scores at each time point.

	Preoperative	3 Months	6 Months	12 Months	Last Follow-up
VAS score for pain					
Group I	3.57 ± 1.87	2.81 ± 1.71	2.02 ± 1.20	2.49 ± 2.45	1.57 ± 0.98
Group II	2.96 ± 2.05	2.58 ± 1.74	2.58 ± 1.67	1.24 ± 1.32	1.33 ± 1.34
*p* value	0.06	0.59	0.15	0.11	0.65
ASES score					
Group I	55.86 ± 13.38	64.63 ± 19.18	71.90 ± 12.07	71.25 ± 21.72	79.94 ± 11.17
Group II	54.67 ± 22.33	65.80 ± 16.61	73.71 ± 15.51	83.13 ± 12.89	82.83 ± 11.94
*p* value	0.80	0.83	0.70	0.047	0.45
CS					
Group I	62.50 ± 16.38	63.38 ± 15.15	75.63 ± 9.15	74.13 ± 13.62	81.56 ± 11.25
Group II	60.08 ± 19.18	66.00 ± 16.20	77.42 ± 12.14	80.97 ± 14.42	82.94 ± 7.17
*p* value	0.49	0.98	0.54	0.25	0.72
KSS					
Group I	60.73 ± 14.51	62.43 ± 12.94	73.52 ± 12.45	70.60 ± 15.72	81.31 ± 16.87
Group II	53.38 ± 19.71	63.48 ± 15.85	78.43 ± 12.47	80.67 ± 14.30	85.69 ± 10.21
*p* value	0.24	0.93	0.12	0.048	0.33

**Table 6:** Repair integrity using Sugaya’s classification at 1 year postoperatively.

	Group I (*n* = 31)	Group II (*n* = 41)
Type I	2	6
Type II	7	10
Type III	13	17
Type IV	2	5
Type V	7	3

## Data Availability

Data are contained within the article.

## References

[1] Bedi A., Dines J., Warren R.F., Dines D.M. (2010). Massive tears of the rotator cuff. J. Bone Jt. Surg. Am..

[2] Kim S.J., Kim Y.H., Chun Y.M. (2014). Arthroscopic Partial Repair of Massive Contracted Rotator Cuff Tears. Clin. Shoulder Elb..

[3] Rho J.Y., Kwon Y.S., Choi S. (2019). Current Concepts and Recent Trends in Arthroscopic Treatment of Large to Massive Rotator Cuff Tears: A Review. Clin. Shoulder Elb..

[4] Burkhart S.S. (1994). Reconciling the paradox of rotator cuff repair versus debridement: A unified biomechanical rationale for the treatment of rotator cuff tears. Arthroscopy.

[5] Burkhart S.S. (1991). Arthroscopic treatment of massive rotator cuff tears. Clinical results and biomechanical rationale. Clin. Orthop. Relat. Res..

[6] Kim S.J., Lee I.S., Kim S.H., Lee W.Y., Chun Y.M. (2012). Arthroscopic partial repair of irreparable large to massive rotator cuff tears. Arthroscopy.

[7] Shon M.S., Koh K.H., Lim T.K., Kim W.J., Kim K.C., Yoo J.C. (2015). Arthroscopic Partial Repair of Irreparable Rotator Cuff Tears: Preoperative Factors Associated with Outcome Deterioration over 2 Years. Am. J. Sports Med..

[8] Han S.Y., Lee T.Q., Wright D.J., Park I.J., Mauro M., McGarry M.H., Lee H.J., Kim Y.S. (2020). Effect of biceps rerouting technique to restore glenohumeral joint stability for large irreparable rotator cuff tears: A cadaveric biomechanical study. J. Shoulder Elb. Surg..

[9] Veen E.J.D., Stevens M., Diercks R.L. (2018). Biceps Autograft Augmentation for Rotator Cuff Repair: A Systematic Review. Arthroscopy.

[10] Park S.R., Sun D.H., Kim J., Lee H.J., Kim J.B., Kim Y.S. (2018). Is augmentation with the long head of the biceps tendon helpful in arthroscopic treatment of irreparable rotator cuff tears?. J. Shoulder Elb. Surg..

[11] Lin J., Qi W., Liu Z., Chen K., Li X., Yan Y., Xu X., Xue X., Yang Y., Pan X. (2019). An arthroscopic technique for full-thickness rotator cuff repair by transposition of the long head of biceps. Orthop. Traumatol. Surg. Res..

[12] Han F., Kong C.H., Hasan M.Y., Ramruttun A.K., Kumar V.P. (2019). Superior capsular reconstruction for irreparable supraspinatus tendon tears using the long head of biceps: A biomechanical study on cadavers. Orthop. Traumatol. Surg. Res..

[13] El-Shaar R., Soin S., Nicandri G., Maloney M., Voloshin I. (2018). Superior Capsular Reconstruction with a Long Head of the Biceps Tendon Autograft: A Cadaveric Study. Orthop. J. Sports Med..

[14] Kim Y.S., Lee H.J., Park I., Sung G.Y., Kim D.J., Kim J.H. (2018). Arthroscopic In Situ Superior Capsular Reconstruction Using the Long Head of the Biceps Tendon. Arthrosc. Tech..

[15] Rhee S.M., Youn S.M., Park J.H., Rhee Y.G. (2021). Biceps Rerouting for Semirigid Large-to-Massive Rotator Cuff Tears. Arthroscopy.

[16] Yoo J.C., Ahn J.H., Koh K.H., Lim K.S. (2009). Rotator cuff integrity after arthroscopic repair for large tears with less-than-optimal footprint coverage. Arthroscopy.

[17] Sugaya H., Maeda K., Matsuki K., Moriishi J. (2005). Functional and structural outcome after arthroscopic full-thickness rotator cuff repair: Single-row versus dual-row fixation. Arthroscopy.

[18] Xu J., Li Y., Zhang X., Han K., Ye Z., Wu C., Jiang J., Yan X., Su W., Zhao J. (2022). The Biomechanical and Histological Processes of Rerouting Biceps to Treat Chronic Irreparable Rotator Cuff Tears in a Rabbit Model. Am. J. Sports Med..

[19] Zabrzyński J., Gagat M., Łapaj Ł., Paczesny Ł., Yataganbaba A., Szwedowski D., Huri G. (2021). Relationship between long head of the biceps tendon histopathology and long-term functional results in smokers. A time to reevaluate the Bonar score?. Ther. Adv. Chronic Dis..

[20] Mori D., Funakoshi N., Yamashita F. (2013). Arthroscopic surgery of irreparable large or massive rotator cuff tears with low-grade fatty degeneration of the infraspinatus: Patch autograft procedure versus partial repair procedure. Arthroscopy.

[21] Wellmann M., Lichtenberg S., da Silva G., Magosch P., Habermeyer P. (2013). Results of arthroscopic partial repair of large retracted rotator cuff tears. Arthroscopy.

[22] Koh K.H., Lim T.K., Park Y.E., Lee S.W., Park W.H., Yoo J.C. (2014). Preoperative factors affecting footprint coverage in rotator cuff repair. Am. J. Sports Med..

[23] Lee Y.S., Jeong J.Y., Park C.D., Kang S.G., Yoo J.C. (2017). Evaluation of the Risk Factors for a Rotator Cuff Retear after Repair Surgery. Am. J. Sports Med..

[24] Kim J.H., Lee H.J., Park T.Y., Lee J.U., Kim Y.S. (2020). Preliminary Outcomes of Arthroscopic Biceps Rerouting for the Treatment of Large to Massive Rotator Cuff Tears. J. Shoulder Elb. Surg..

[25] Zabrzynski J., Huri G., Gagat M., Lapaj L., Yataganbaba A., Szwedowski D., Askin M., Paczesny L. (2021). The Impact of Smoking on Clinical Results following the Rotator Cuff and Biceps Tendon Complex Arthroscopic Surgery. J. Clin. Med..

[26] Zabrzynski J., Huri G., Gryckiewicz S., Cetik R.M., Szwedowski D., Lapaj L., Gagat M., Paczesny L. (2020). Biceps Tenodesis versus Tenotomy with Fast Rehabilitation Protocol—A Functional Perspective in Chronic Tendinopathy. J. Clin. Med..

[27] Belk J.W., Kraeutler M.J., Houck D.A., Chrisman A.N., Scillia A.J., McCarty E.C. (2021). Biceps tenodesis versus tenotomy: A systematic review and meta-analysis of level I randomized controlled trials. J. Shoulder Elb. Surg..

